# Systematic Investigation of Immune-Related lncRNA Landscape Reveals a Potential Long Non-Coding RNA Signature for Predicting Prognosis in Renal Cell Carcinoma

**DOI:** 10.3389/fgene.2022.890641

**Published:** 2022-07-04

**Authors:** Kepu Liu, Zhibin Li, Dongli Ruan, Huilong Wang, Wei Wang, Geng Zhang

**Affiliations:** ^1^ Department of Urology, Xijing Hospital, Air Force Military Medical University, Xi’an, China; ^2^ Department of Urology, Xi’an People’s Hospital (Xi’an Fourth Hospital), Xi’an, China; ^3^ Department of Urology and Nephropathy, Xi’an People’s Hospital (Xi’an Forth Hospital), Xi’an, China; ^4^ YuceBio Technology Co., Ltd., Shenzhen, China; ^5^ Department of Urology, Xijing Hospital, Fourth Military Medical University, Xi’an, China

**Keywords:** renal cell carcinoma, long non-coding RNAs, prognostic signature, cancer immunity, immune infiltration

## Abstract

**Background:** Renal cell carcinoma (RCC) is the predominant type of malignant tumor in kidney cancer. Finding effective biomarkers, particularly those based on the tumor immune microenvironments (TIME), is critical for the prognosis and diagnosis of RCC. Increasing evidence has revealed that long non-coding RNAs (lncRNAs) play a crucial role in cancer immunity. However, the comprehensive landscape of immune infiltration-associated lncRNAs and their potential roles in the prognosis and diagnosis of RCC remain largely unexplored.

**Methods:** Based on transcriptomic data of 261 RCC samples, novel lncRNAs were identified using a custom pipeline. RCC patients were classified into different immune groups using unsupervised clustering algorithms. Immune-related lncRNAs were obtained according to the immune status of RCC. Competing endogenous RNAs (ceRNA) regulation network was constructed to reveal their functions. Expression patterns and several tools such as miRanda, RNAhybrid, miRWalk were used to define lncRNAs-miRNAs-mRNAs interactions. Univariate Cox, LASSO, and multivariate Cox regression analyses were performed on the training set to construct a tumorigenesis-immune-infiltration-related (TIR)-lncRNA signature for predicting the prognosis of RCC. Independent datasets involving 531 RCC samples were used to validate the TIR-lncRNA signature.

**Results:** Tens of thousands of novel lncRNAs were identified in RCC samples. Comparing tumors with controls, 1,400 tumorigenesis-related (TR)-lncRNAs, 1269 TR-mRNAs, and 192 TR-miRNAs were obtained. Based on the infiltration of immune cells, RCC patients were classified into three immune clusters. By comparing immune-high with immune-low groups, 241 TIR-lncRNAs were identified, many of which were detected in urinary samples. Based on lncRNA-miRNA-mRNA interactions, we constructed a ceRNA network, which included 25 TR-miRNAs, 28 TIR-lncRNAs, and 66 TIR-mRNAs. Three TIR lncRNAs were identified as a prognostic signature for RCC. RCC patients in the high-risk group exhibited worse OS than those in the low-risk group in the training and testing sets (*p* < 0.01). The AUC was 0.9 in the training set. Univariate and multivariate Cox analyses confirmed that the TIR-lncRNA signature was an independent prognostic factor in the training and testing sets.

**Conclusion:** Based on the constructed immune-related lncRNA landscape, 241 TIR-lncRNAs were functionally characterized, three of which were identified as a novel TIR-lncRNA signature for predicting the prognosis of RCC.

## Introduction

Kidney cancer is among the most common malignant tumors worldwide, with an estimated nearly 0.4 million new cases (2.2%), and the leading cause of cancer-related deaths (was nearly 0.2 million; 1.8% of the total cancer-related deaths) according to the latest GLOBOCAN 2020 data (Sung et al., 2021). Renal cell carcinoma (RCC) is the predominant type of malignant tumor affecting the kidney, accounting for over 90% of malignant tumors in this organ (Moch et al., 2016). Compared to early or localized RCC, advanced disease has a poor prognosis, with a 5-years survival rate of less than 12% (Atkins and Tannir, 2018; Rao et al., 2018). Recent studies have reported several prognostic models for RCC. However, the Area Under Curve (AUC) values were all less than 0.83 (Qi-Dong et al., 2020; Ma et al., 2021; Sun et al., 2021; Yu et al., 2021). Therefore, a more efficient model is urgently needed for predicting the prognosis of RCC.

Long non-coding RNAs (lncRNAs) are longer than 200 nucleotides and can not encode proteins. Recent studies reported that lncRNAs are involved in multiple biological and cancer-related processes, including tumorigenesis, progression, and metastasis (Moran et al., 2012; Bhan et al., 2017; Peng et al., 2017; Yao et al., 2019; Bao et al., 2020). Increasing evidence have revealed that lncRNAs play crucial roles in cancer immunity (Denaro et al., 2019; Wu et al., 2020). However, the comprehensive landscape of immune infiltration-associated lncRNAs and their potential roles in the prognosis and diagnosis of RCC remain largely unexplored.

Based on raw transcriptomic data from RCC patients, we aim to construct a comprehensive lncRNA landscape for RCC, characterize the regulation in tumor immune microenvironments (TIME), and construct a prognostic signature for RCC.

## Materials and Methods

### Data Sources and Expression Analysis

In our study, a total of 303 data from RCC patients were downloaded from the Gene Expression Omnibus database (GEO, http://www.ncbi.nlm.nih.gov/geo), including tissue and urinary raw transcriptomics data, tissue miRNA data, and clinical information. 261 tissue raw transcriptomics data were used to identify novel lncRNAs. Tissue transcriptomics data and miRNAs data were used to calculate tumorigenesis-related (TR-) lncRNAs, TR-mRNAs, and TR-miRNAs by comparing tumors with controls. All tumor samples were used to investigate the immune infiltration, classify immune groupings, identify tumorigenesis-immune-infiltration-related (TIR)-lncRNAs and TIR-mRNAs. Raw transcriptomics data from urinary samples were used to assess the release of tumor TIR-lncRNAs into the urine in RCC. Tumor transcriptomics data with survival information was regarded as the training set to construct the prognostic model based on TIR-lncRNAs. The detailed information of GEO datasets in our study were shown in Table1.

**TABLE 1:** Detailed information of GEO datasets.

GEO	Source	Data	Tumors	Controls
GSE167573	Tissue	Raw transcriptome data with survival information	62	14
GSE126964	Tissue	Raw transcriptome data	55	11
GSE151419	Tissue	Raw transcriptome data	58	17
GSE143630	Tissue	Raw transcriptome data	44	-
GSE151423	Tissue	miRNA	26	6
GSE125442	Urine	Raw transcriptome data	10	-

Besides, we also collected 531 data from kidney renal clear cell carcinoma patients which were downloaded from The Cancer Genome Atlas (TCGA) database, including tumor transcriptomics data and clinical information. These data were independent of the training set, which was regarded as the testing set to validate the prognostic model. TPM (transcripts per million) was used to normalize the gene expression level, and log2 transformed (log2 (TPM+1)).

Raw transcriptome data were analyzed by FastQC v0.11.3 with default parameters (http://www.bioinformatics.babraham.ac.uk/projects/fastqc/) and removed the adapters and low-quality sequences by TrimGalore-0.6.0 with default parameters (https://www.bioinformatics.babraham.ac.uk/projects/trim_galore/). Clean reads were mapped by using STAR v.2.7.8a (Dobin et al., 2013; Dobin and Gingeras, 2015) (set the *twopassMode* as Basic), *de novo* assembled by using StringTie v2.1.6, and merged by using the cuffmerge function of Cufflinks v2.2.1 (Trapnell et al., 2010). The human reference genome version hg38/GRCh38 was utilized. Reads counts and TPM values were calculated by Kallisto v.0.46.2 (Bray et al., 2016) with default parameters.

### Identification of Novel lncRNAs in RCC

Based on assembled transcripts, we compared it with GENCODE v38 (Frankish et al., 2019) and RefLncRNA (Jiang et al., 2019) genes annotation by using the cuffcompare function of Cufflinks (Trapnell et al., 2010), respectively. The assembled transcripts were classified into four categories according to the “class code” information, including “complete match” (=), “partial match” (j), “contained” (c), and “not match”. Not matched transcripts (class code included “i, x, u”) were further used to identify the reliable novel lncRNAs by the following steps (Luo et al., 2021): ⅰ) transcript length>=200; ⅱ) have more than one exon; ⅲ) recurrence in at least two samples; ⅳ) identified as novel lncRNAs in both CPC2(Coding Potential Calculator) (Kang et al., 2017) and CNCI (Coding Noncoding Index) (Sun et al., 2013). The final lncRNAs catalog was obtained by combining the RefLncRNA and novel lncRNAs directly.

### Identification of TR-lncRNAs, TR-mRNAs, and TR-miRNAs in RCC

To obtain TR-lncRNAs, TR-mRNAs, and TR-miRNAs, the “DESeq2” package in R was used to analyze the transcripts data and miRNAs data by comparing tumors with controls with the cutoff criteria (adjusted *p*-value < 0.05 and | log2 fold change | >1). Genes with low expression levels (i.e., which were expressed only in one sample and the sum of expression levels of all samples less than 10) were removed from the data.

### Identification of Immune Groups, IR-lncRNAs and TIR-lncRNAs in RCC

Single sample gene set enrichment analysis (ssGSEA) was performed by “GSVA” packages in R to calculate the enrichment scores of 28 types of immune cells in the tumor microenvironment (Hänzelmann et al., 2013; Charoentong et al., 2017). Tumors were further classified into different immune groups by using the unsupervised clustering algorithm (“ConsensusClusterPlus” packages in R). And then ESTIMATE algorithms (“estimate” packages in R) were used to confirm these immune groupings by calculating the immune score, stromal score, and estimate score. By comparing the immune-high group with the immune-low group, IR-lncRNAs were calculated by “DESeq2” with the cutoff criteria (adjusted *p*-value < 0.001 and | log2 fold change | >3). IR-mRNAs were calculated by “DESeq2” with the cutoff criteria (adjusted *p*-value < 0.05 and | log2 fold change | >1). Through the intersection analysis, TIR-lncRNAs and TIR-mRNAs were obtained.

### Construction of ceRNA Network

miRanda (John et al., 2004) (http://www.miRNA.org/) and RNAhybrid (Krüger and Rehmsmeier, 2006) (http://bibiserv.techfak.uni-bielefeld.de/rnahybrid/) was used to predict TIR-lncRNAs and TR-miRNA interactions. ‘-sc’ set as 160 in miRanda and set “-b 1 -e -25 -f 8,12 -u 1 -v 1 -s 3utr_human” in RNAhybrid. The TIR-mRNAs and TR-miRNAs interactions were predicted by miRWalk (Dweep et al., 2011; Dweep and Gretz, 2015) (http://mirwalk.umm.uni-heidelberg.de/). TargetScan (Agarwal et al., 2015) and miRDB (Liu and Wang, 2019; Chen and Wang, 2020) databases were used to confirm this prediction. The “psych” package in R was used to calculate the correlation between lncRNAs and mRNAs. The positive correlated pairs between lncRNA and mRNA were selected with the cutoff criteria (adjusted *p*-value < 0.05 and correlation coefficient >0.65). Based on the miRNA-mRNA, miRNA-lncRNA, and mRNA-lncRNA pairs, the lncRNA–miRNA–mRNA ceRNA network was constructed and visualized by Cytoscape v3.8.2 software (Shannon et al., 2003).

### Investigation of the Releasing of Tumor TIR-lncRNAs Into the Urine

Raw urinary transcriptome data from RCC patients were quality controlled, mapped, *de novo* assembled, and merged using the same methods as tissue transcriptome data. The primary assembled transcripts were used to compare with the TIR-lncRNAs catalog, GENCODE v38 (Frankish et al., 2019), and RefLncRNA (Jiang et al., 2019) genes annotation by using the cuffcompare function of the Cufflinks package, respectively.

### Construction and Validation of the TIR-lncRNA Signature

In the training set, univariate Cox regression, LASSO regression, and multivariate Cox regression analyses were performed by “survival”, “survminer”, and “glmnet” packages in R to screen prognosis-related TIR-lncRNAs and to construct a TIR-lncRNA signature for predicting the prognosis of RCC. *p* < 0.05 was considered to be related to the prognosis. The risk score for each patient was calculated by the following formula. Log2-transformed TPM was used.

$$Risk\;score=\overset{n}{\underset{n=1}{\sum }}\left(Coef_{i}\times \log2\;\mathrm{transformed TP}M_{lncRNA\;i}\right)$$

RCC patients in the training set were divided into high-risk and low-risk groups according to the median value of risk score. Kaplan-Meier (K-M) survival analysis (“survival” and “survminer” packages in R) was performed to compare the survival rate between the high-risk and low-risk groups. Receiver-operating characteristic (ROC) analysis (“pROC” packages in R) was performed to evaluate the sensitivity and specificity of the TIR-lncRNA signature.

In the testing set, the risk score was calculated for each patient by the same formula as the training set. RCC patients in the testing set were divided into high-risk and low-risk groups according to the same cutoff as the training set. The K-M survival analysis was performed to compare the survival rate between the high-risk and low-risk groups.

Univariate and multivariate Cox regression analyses were used to assess whether TIR-lncRNA signature was an independent predictor for RCC patients among other clinical information, including age, gender, tumor size, and cancer stage.

In addition, a nomogram score system was constructed using the “rms” and “survival” packages in R, based on the TIR-lncRNA signature, age, gender, tumor size, and pathological stage in the training set, to predict the survival of RCC patients. Each variable was allocated a point in the nomogram score system, adding up to a total point for each sample that predicts 1-, 3-, and 5-years survival (Iasonos et al., 2008).

### Gene Functional Enrichment Analysis

To explore the functions of TR-lncRNAs and TIR-lncRNAs, functional enrichment analyses were conducted using the online databases KOBAS 3.0 (http://kobas.cbi.pku.edu.cn) and “Metascape” (Zhou et al., 2019) (http://metascape.org).

### Statistical Analysis

All statistical analyses were conducted using the R software version 4.1.1. Forest-plot was plotted by “forestplot” packages in R. Upset plot was plotted by “ComplexHeatmap” packages in R. All comparisons for continuous variables were performed using the two-tailed Wilcoxon test for two groups. For categorical variables, Pearson’s Chi-squared test was used. The FDR method in R was used to adjust the *p*-value outputted in multiple comparisons. *p*-value or adjusted *p*-values < 0.05 were considered as the significance level.

## Results

### Construction of a Comprehensive lncRNA Catalog for RCC Patients

**FIGURE 1:**
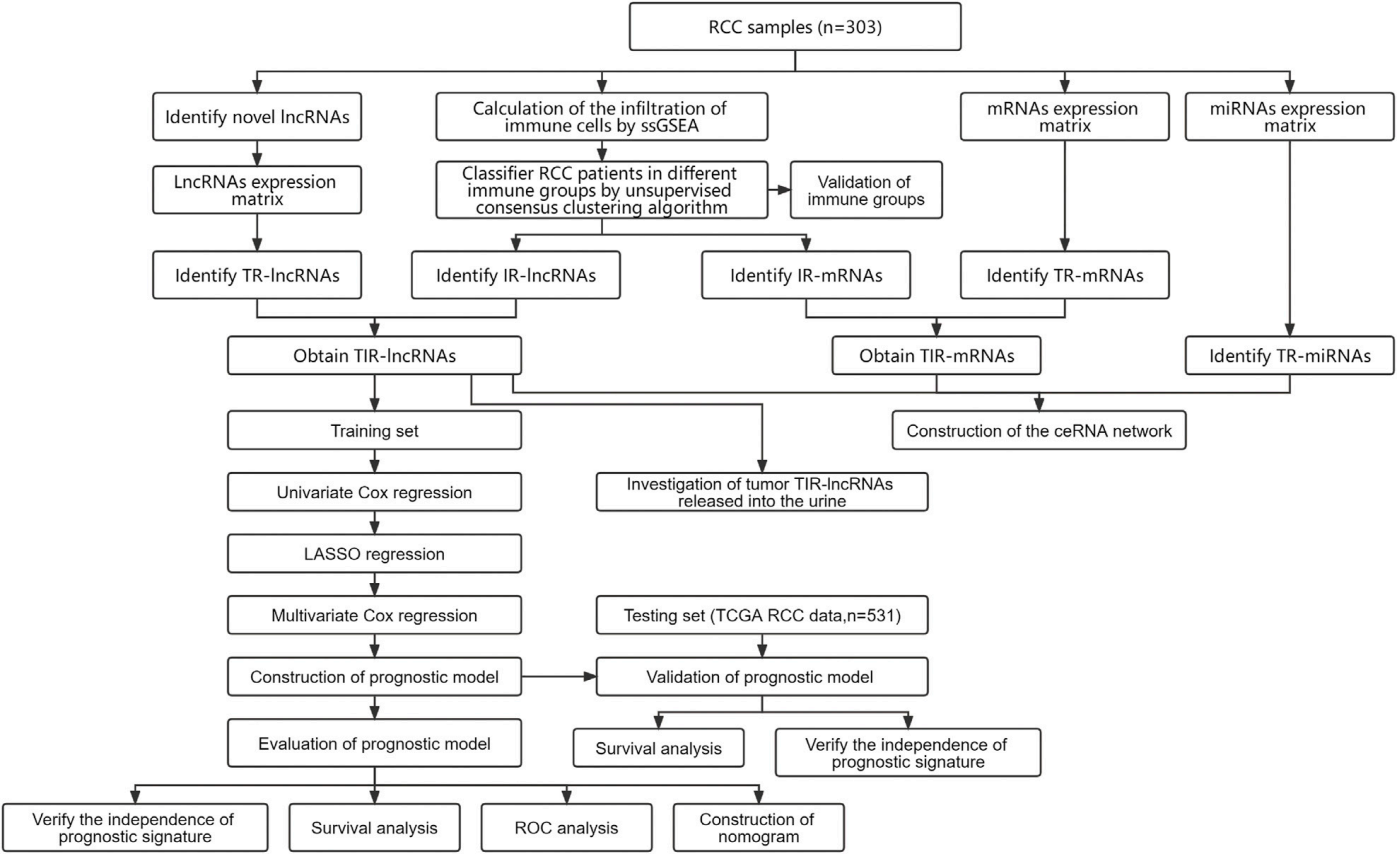
The overall workflow and study design showed the process of identifying novel lncRNAs, identifying TIR-lncRNAs, TIR-mRNAs, and TR-miRNAs, constructing ceRNA network, assessing tumor lncRNAs shedding into the urine, constructing and validating the 3-TIR-lncRNAs classifiers to predict the prognosis of RCC. RCC, renal cell carcinoma; lncRNAs, long noncoding RNAs; ROC, receiver-operating characteristic.

**FIGURE 2:**
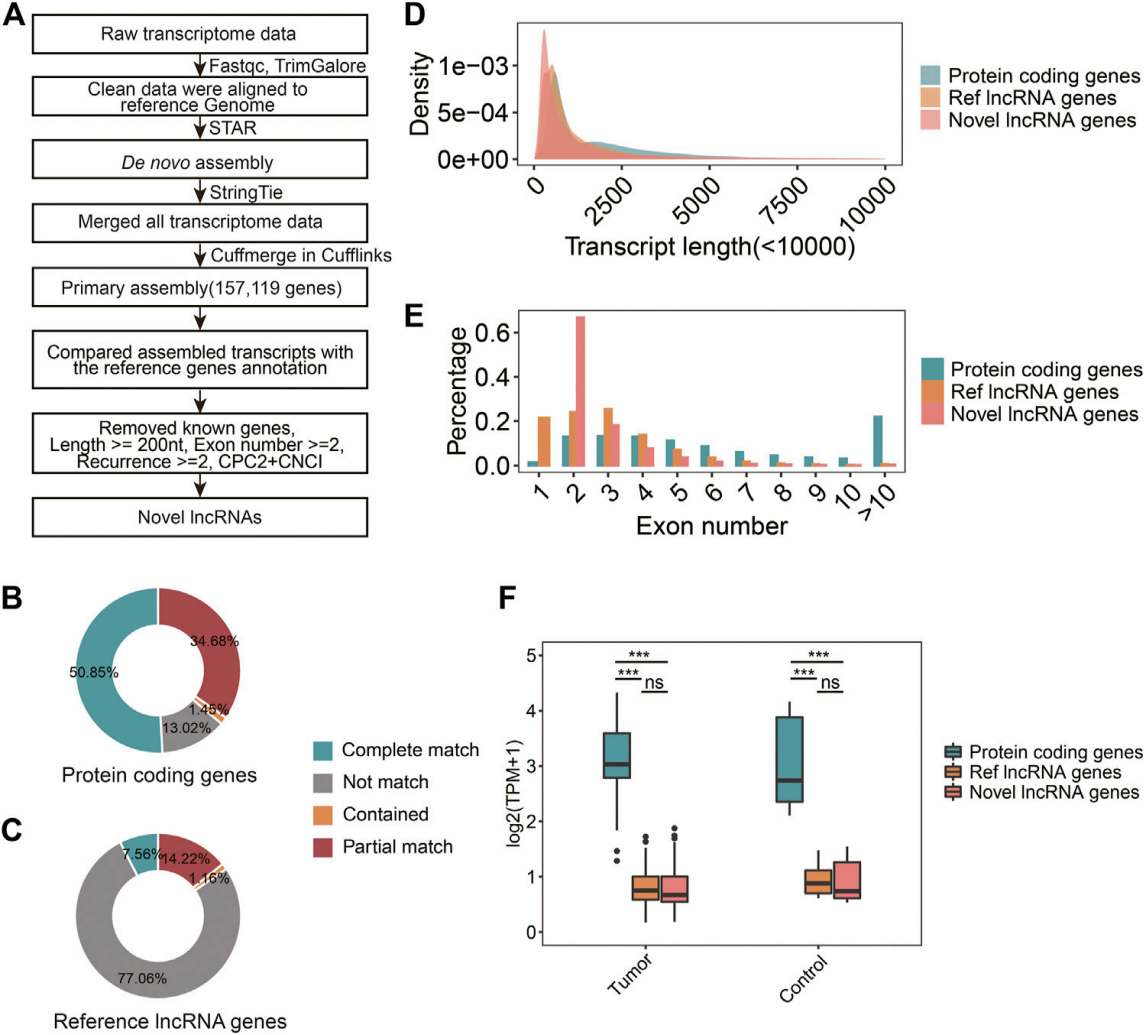
Identification and characterization of novel lncRNA. **(A)** The identification process of novel lncRNAs. **(B)** The statistics of assembled transcripts matched to GENCODE v38 genes annotation. **(C)** The statistics of assembled transcripts matched to RefLncRNA genes annotation. **(D)** Density diagrams showed the transcript length in protein-coding genes, reference lncRNAs, and novel lncRNAs **(E)** Bar plot showed exon numbers in protein-coding genes, reference lncRNAs, and novel lncRNAs. **(F)** Boxplot showed transcript expression levels of protein-coding genes, reference lncRNAs, and novel lncRNAs in tumors and controls.

In order to systematically investigate lncRNAs and their roles in RCC immunity, raw transcriptome data from RCC tissues were used to identify novel lncRNAs. The workflow was shown in Figure 1. After quality control, reads alignment, *de novo* transcriptome assembly, and merging, 157,119 primary genes were obtained (Figure 2A). To assess the accuracy of the assembly results, comparative analysis was performed using reference protein-coding genes and RefLncRNA genes annotation. More than 86% of the protein-coding genes were verified, and over 50% were completely matched (Figure 2B). In comparison, only 22.94% of the reference lncRNAs were verified (Figure 2C). Based on the primary assembled transcripts that did not match the reference genes, a custom pipeline was used to identify reliable lncRNAs (see Methods 2.4). Finally, 44,507 novel lncRNA genes were identified (Figure 2A).

To further characterize the novel lncRNAs, we analyzed their transcript lengths, exon numbers, and expression profiles. The mean transcript length was 1.4 k nucleotides and exon numbers mainly ranged from 2-5, which were close to reference lncRNAs (Figures 2D,E). These findings are consistent with those of previous studies (Bo et al., 2021; Wang et al., 2021). The genes expression levels of novel lncRNAs were significantly lower than protein-coding genes in both tumors and controls (*p* < 0.001, Figure 2F). There was no significant difference in genes expression levels between the novel and reference lncRNAs (*p* > 0.05, Figure 2F).

### Identification of TR-lncRNAs

**FIGURE 3:**
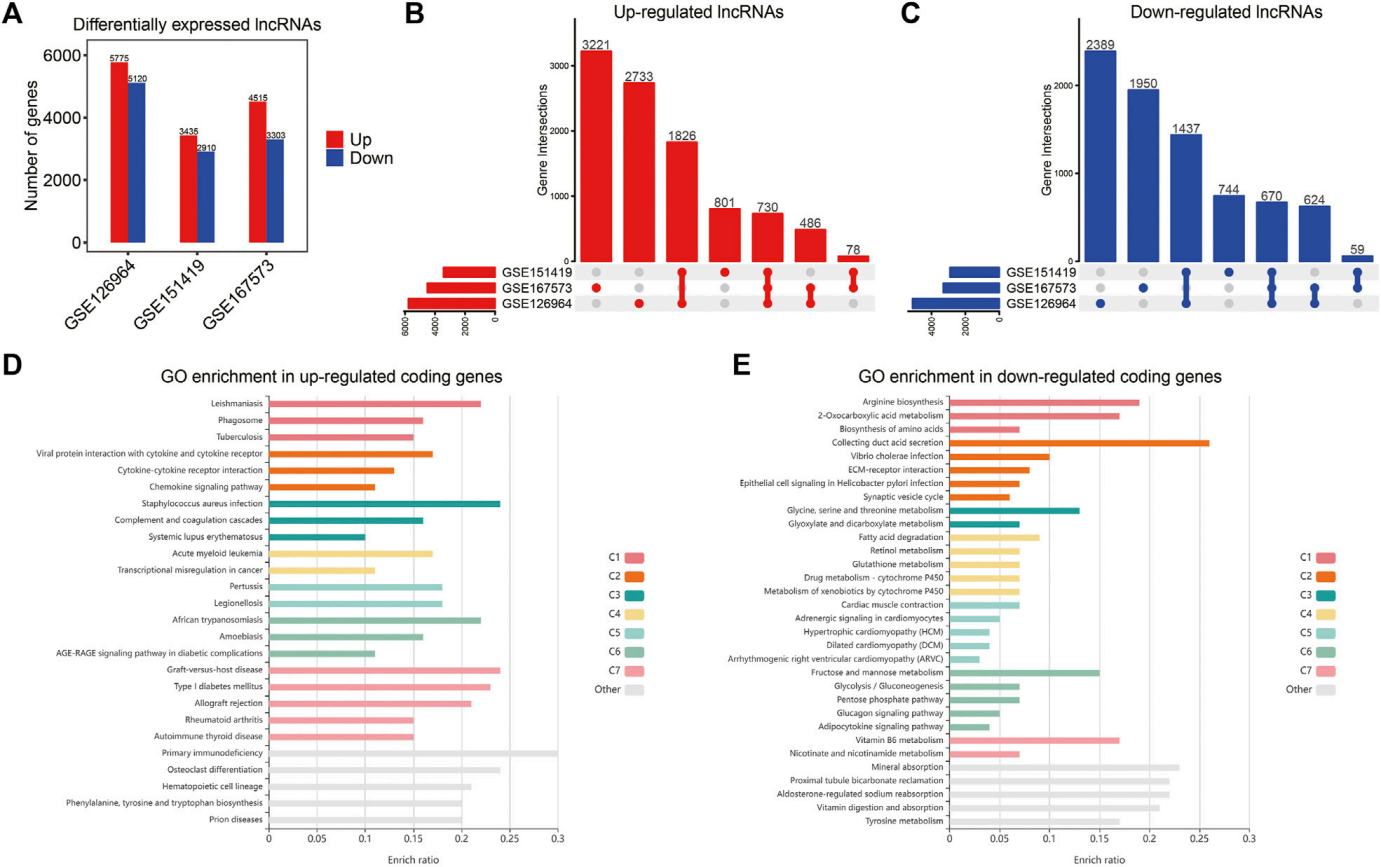
Identification of TR-lncRNAs by comparing tumors with controls in RCC. **(A)** Histogram of TR-lncRNAs number in three GEO datasets. **(B)** Upset plots of the distribution of upregulated lncRNAs in each dataset. **(C)** Upset plots of the distribution of downregulated lncRNAs in each dataset. **(D)** Bar plot showed GO enrichment pathways of upregulated genes. **(E)** Bar plots showed GO enrichment pathways of downregulated genes.

Based on the integrated lncRNA expression matrix, we calculated the TR-lncRNAs between RCC tumors and controls. In total, 1,400 TR-lncRNAs (730 upregulated and 670 downregulated) were identified, including 520 novel lncRNAs (Figures 3A–C, Supplementary Table S1). Similarly, 1,269 TR-mRNAs (715 upregulated and 554 downregulated) were identified (Supplementary Figures S1A–C, Supplementary Table S2). To investigate the functions of the TR-lncRNAs, functional enrichment analysis of the TR-mRNAs was performed. Upregulated genes were mainly enriched in cytokine, chemokine, and immune-associated pathways, including cytokine-cytokine receptor interaction, chemokine signaling pathway, and primary immunodeficiency (Figure 3D). In comparison, the downregulated genes were mainly enriched in metabolism-associated pathways, including glycine, serine and threonine metabolism, and fatty acid metabolism (Figure 3E).

### Immune Infiltration Analysis and Identification of TIR-lncRNAs

**FIGURE 4:**
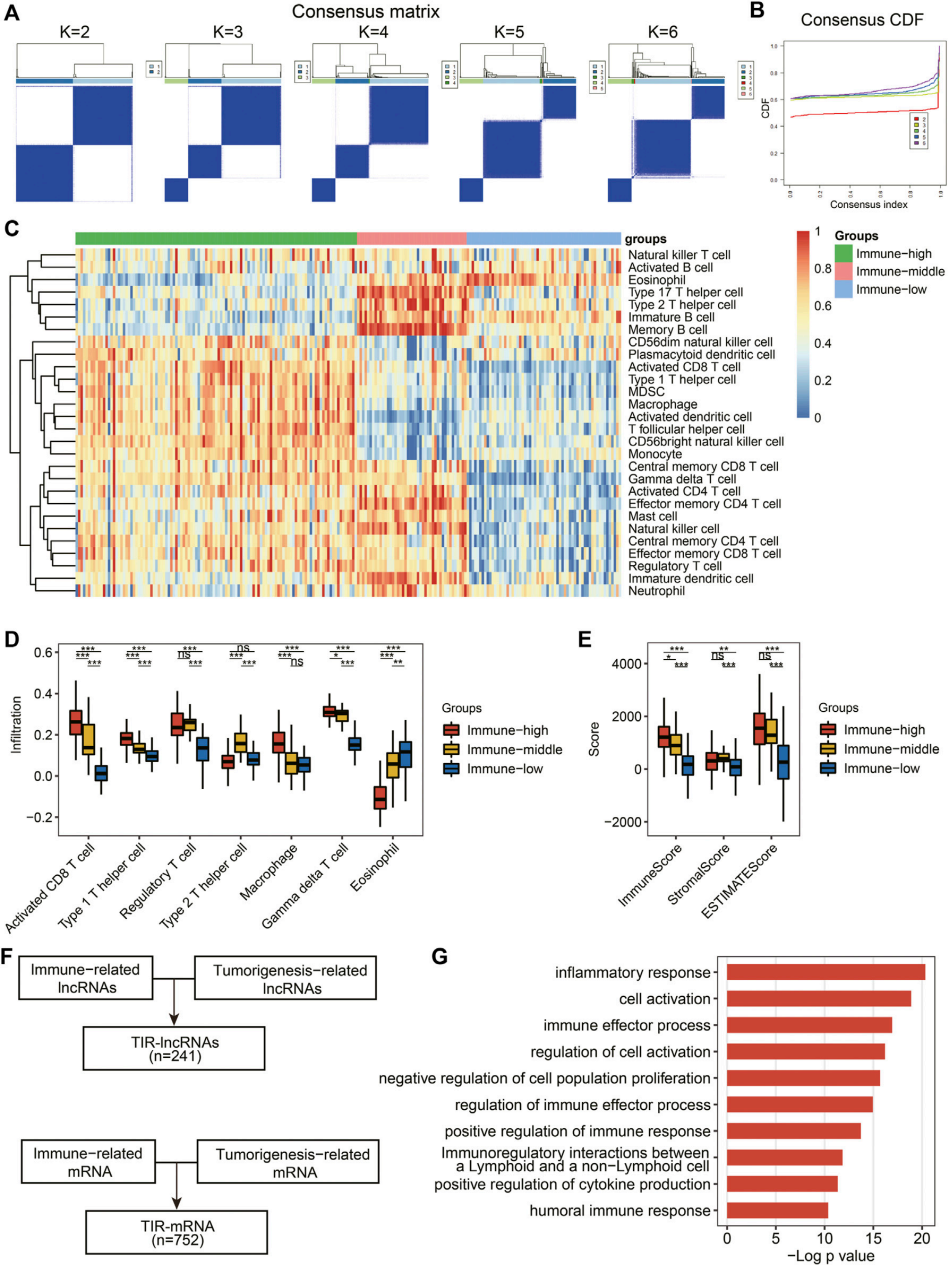
Identification of TIR-lncRNAs and functional enrichment. **(A)** Sample clustering heatmap for k = 2 to 6, respectively. **(B)** The cumulative distribution function (CDF) plots for k = 2 to 6. **(C)** Heatmap of normalized enrichment scores for infiltration of 28 immune-cell types. **(D)** Comparisons among the immune-high group, the immune-middle group, and the immune-low group for seven immune-cell types. **(E)** Comparisons among the immune-high group, the immune-middle group, and the immune-low group for immune score, stromal score, and estimate score. **(F)** Identification of TIR-lncRNAs and TIR-mRNAs. **(G)** Bar plots showed the main GO enrichment pathways of TIR-lncRNAs.

To further explore immune infiltration-related lncRNAs and their roles in the tumor microenvironment, we first calculated the enrichment scores of 28 immune-cell types in each patient by ssGSEA. Based on immune infiltration, an unsupervised clustering algorithm was utilized to classify the RCC patients into three clusters (Figures 4A–C). When k = 3, the classification was more reliable than others (Figures 4A,B). The heatmap showed normalized enrichment scores for the infiltration of 28 immune-cell types in each patient (Figure 4C). Compared with the immune-low group, the immune-high group showed an overall significantly higher degree of infiltration of immune cells, including activated CD8 T cells, T-helper cells type 1 (Th1), regulatory T cells, macrophages, and gamma delta T cells (Figure 4D, Supplementary Figure S2). Similarly, the immune-middle group exhibited a significantly higher degree of infiltration of immune cells than those in the immune-low group (Figure 4D). Interestingly, unlike other immune-cell types, T-helper cell type 2 (Th2) showed a higher degree of infiltration in the immune-middle group than that in the immune-high and immune-low groups (Figure 4D). Eosinophils exhibited a lower degree of infiltration in the immune-high group than that in the immune-middle and immune-low groups (Figure 4D). These findings may be related to the function of eosinophils recruited by Th2 in pathways associated with allergic reactions and inflammatory responses (Maggi, 1998). Immune grouping was confirmed by comparing their immune, stromal, and estimate scores. The scores of the immune-high and immune-middle groups were significantly higher than those of the immune-low group (Figure 4E). The immune-high group had a significantly higher immune score than the immune-middle group (Figure 4E). These findings suggested that immune grouping could be used for subsequent analyses.

Integrative analysis of genes related to immune groups and tumorigenesis revealed 241 TIR-lncRNAs and 752 TIR-mRNAs (Figure 4F, Supplementary Tables S3, S4). TIR-lncRNAs were primarily located on autosomal chromosomes and less frequently on X chromosomes (Supplementary Figure S3). Interestingly, no TIR-lncRNAs were present on the Y chromosome (Supplementary Figure S3). As expected, the predominately enriched pathways of TIR-lncRNAs were involved in immune response- and tumorigenesis-associated pathways according to GO enrichment analysis (Figure 4G, Supplementary Table S5).

### Immune-Related ceRNA Network Construction

**FIGURE 5:**
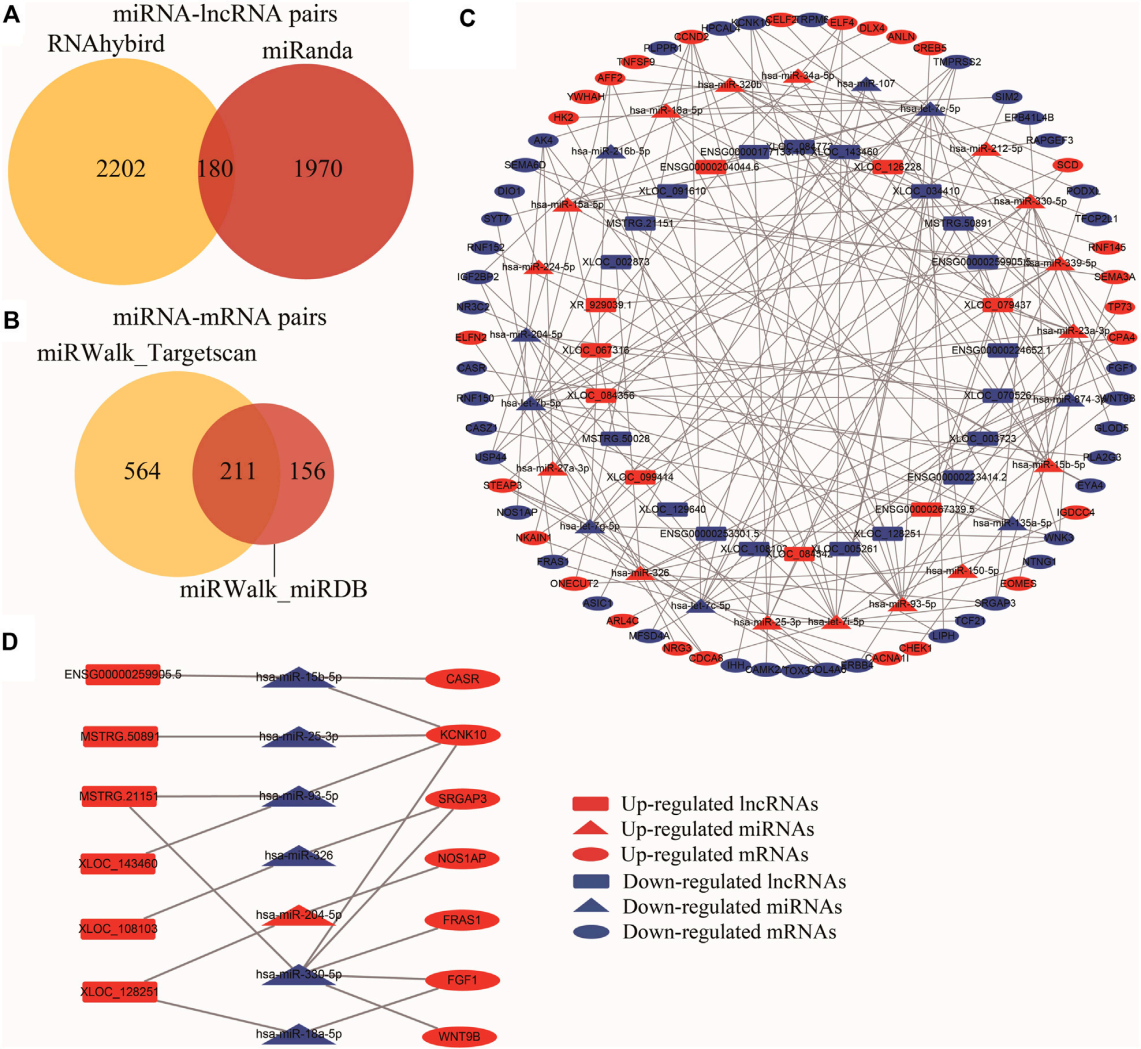
Construction of immune-associated ceRNA network. **(A)** Venn diagram showed the overlapped miRNA-lncRNAs pairs predicted by miRanda and RNAhybrid. **(B)** Venn diagram showed the overlapped miRNA-mRNAs pairs predicted by miRWalk, Targetscan, and miRDB database. **(C)** The ceRNA network consists of 28 TIR-lncRNAs, 25 TR-miRNAs, and 66 mRNAs. LncRNAs, miRNAs, and mRNAs are respectively represented by rectangles, triangles, and ellipses. The red color represented upregulated genes, and the blue color represented downregulated genes in the tumor tissues relative to control tissues. **(D)** The candidate relevant RNAs were further screened based on the correlation between lncRNAs and mRNAs.

To unveil the potential regulatory roles of the 241 TIR-lncRNAs, we constructed a lncRNA/miRNA/mRNA ceRNA network. First, 192 miRNAs, including 88 upregulated and 104 downregulated miRNAs, were identified by comparing RCC tumors with controls, (Supplementary Figure S4, Supplementary Table S6). The RNAhybrid and miRanda databases were used to predict the interactions between the 192 TR-miRNAs and 241 TIR-lncRNAs, revealing 180 miRNA-lncRNA pairs (Figure 5A), including 77 miRNAs and 68 lncRNAs. The miRwalk database was used to predict the interactions between 192 TR-miRNAs and 752 TIR-mRNAs, and the TargetScan and miRDB databases were used to confirm these interactions. In total, 211 miRNA-mRNA pairs were identified (Figure 5B), including 57 miRNAs and 93 mRNAs. Subsequently, the miRNA-lncRNA and miRNA-mRNA pairs were used to construct the lncRNAs-miRNA-mRNA ceRNA network, which included 25 miRNAs (16 upregulated and 9 downregulated), 28 lncRNAs (9 upregulated and 19 downregulated), and 66 mRNAs (26 upregulated and 40 downregulated) (Figure 5C). Next, these screened lncRNAs were used to survey relevant mRNAs based on their correlations. Based on the correlation between lncRNAs and mRNAs, 6 lncRNAs, 7 miRNAs, and 7 mRNAs were identified as candidate relevant RNAs (Figure 5D). GO enrichment analysis showed that the ceRNA network was involved in pathways associated with kidney morphogenesis and the regulation of ion transport.

### A Large Part of Tumor TIR-lncRNAs Can Be Released Into the Urine in RCC

**FIGURE 6:**
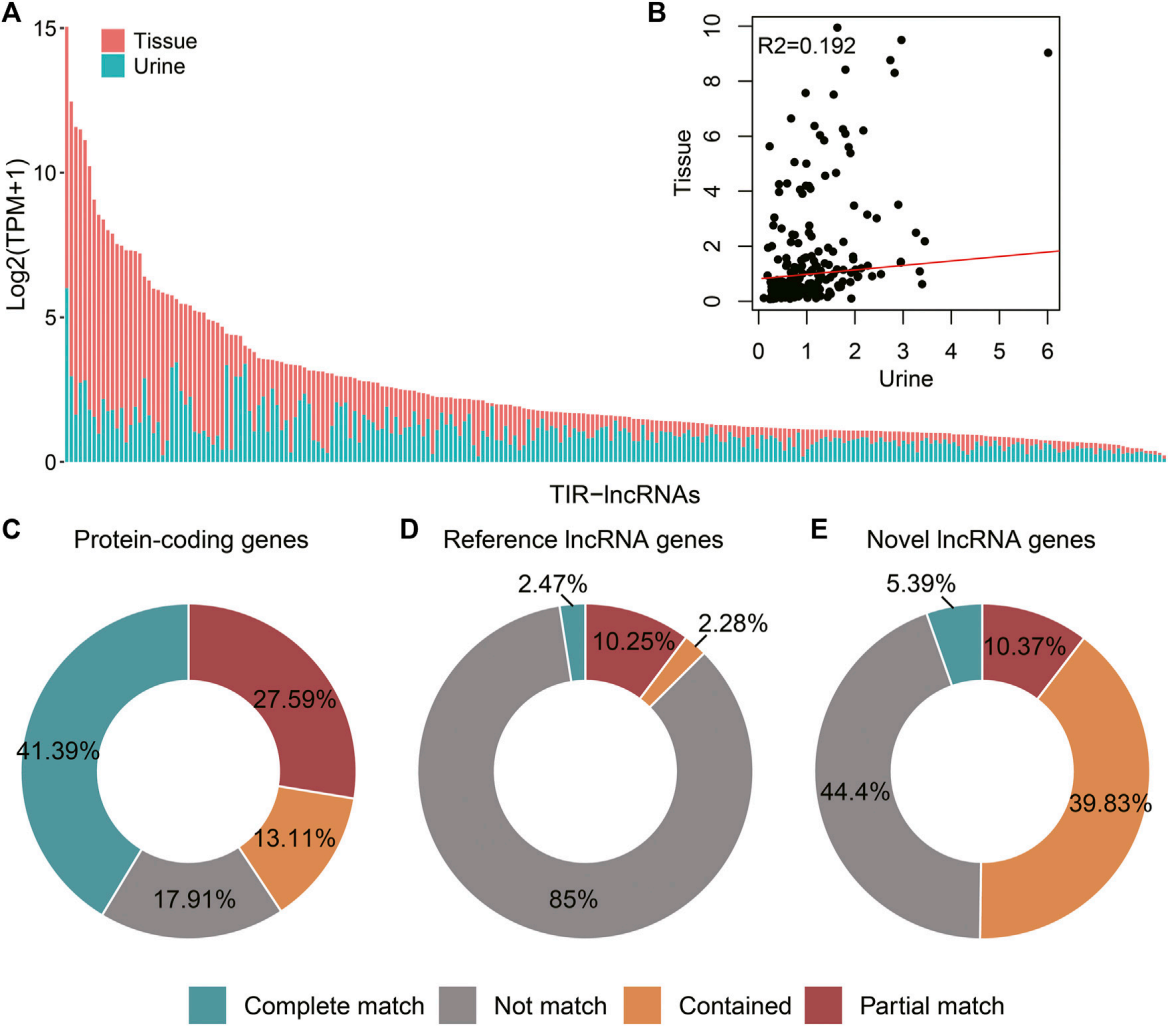
Assessment of tumor TIR-lncRNAs releasing into the urine in RCC. **(A)** Bar plots showed the expression level (log2 transformed TPM) of TIR-lncRNAs in tissue samples and urine samples. **(B)** Scatter plots showed the correlation of TIR-lncRNAs between tissue samples and urine samples. **(C)** The statistics of assembled urinary transcripts matched to GENCODE v38 genes annotation. **(D)** The statistics of assembled urinary transcripts matched to RefLncRNA genes annotation. **(E)** The statistics of assembled urinary transcripts matched to 241 TIR-lncRNAs annotation.

Raw transcript data from RCC urinary samples were analyzed to assess whether TIR-lncRNAs are released into urine. All TIR-lncRNAs were detected in urine, although a large proportion showed low expression levels (Figure 6A). TIR-lncRNAs showed a positive correlation between urinary and tissue samples (*r* ^2^ = 0.192, *p* = 9.987e-13. Figure 6B). To further evaluate the transcript features in the urine, we performed *de novo* assembly analysis. A total of 1,554,672 genes were primary assembled in urine, which were compared with reference genes annotation and catalog of 241 TIR-lncRNAs. Over 82% of the protein-coding genes and 15% of the reference lncRNAs were verified (Figures 6C,D). Moreover, more than 55% of the TIR-lncRNAs were verified, 5.39% were completely matched, 10.37% were partially matched, and 39.83% were contained (Figure 6E).

### Efficient TIR-lncRNA Signature for Predicting the Prognosis of RCC

**FIGURE 7:**
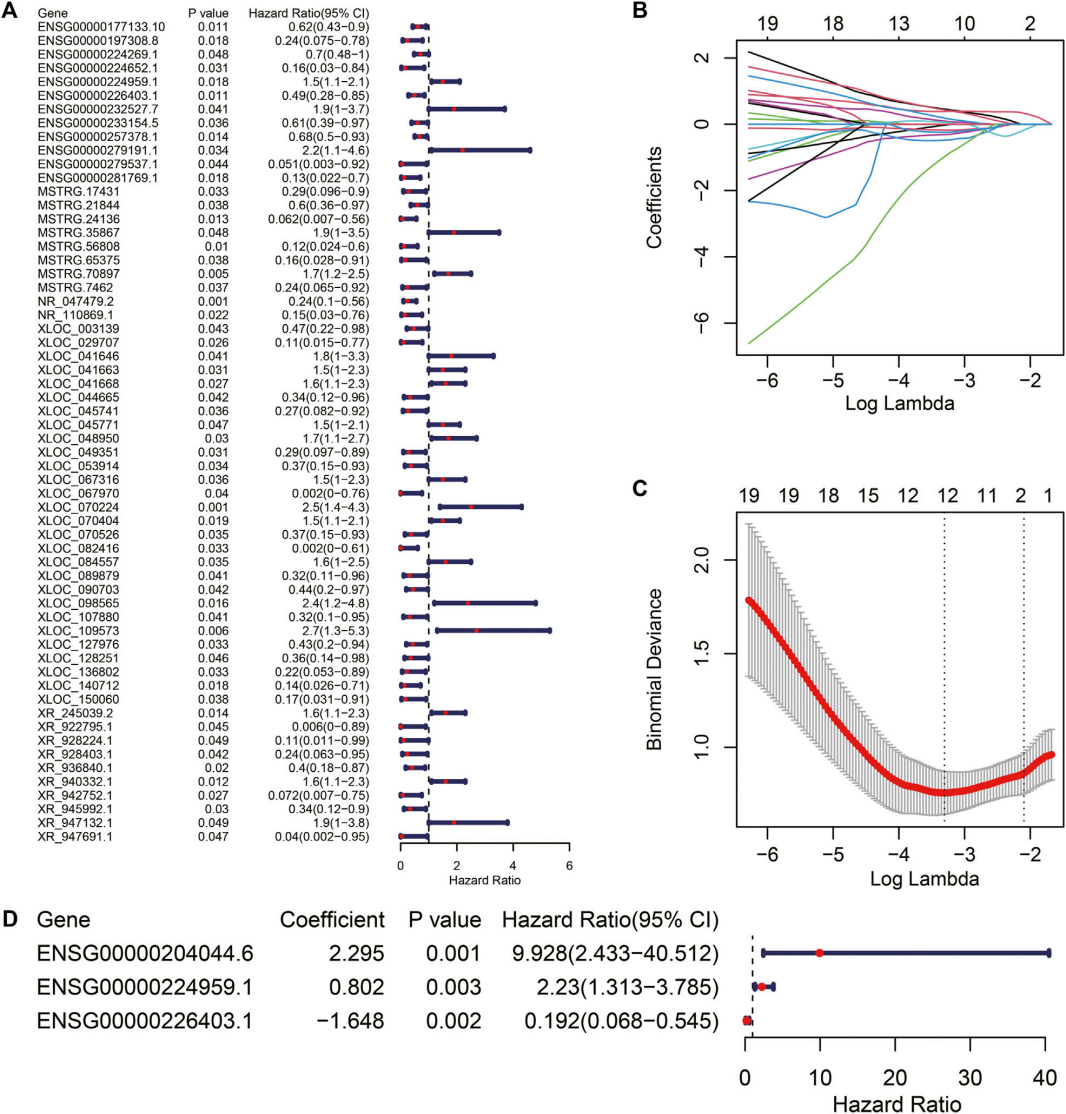
Construction of TIR-lncRNA signature in RCC. **(A)** The Forest plot showed the *p*-value, HR, and 95%CI of prognosis-related TIR-lncRNAs calculated by univariate Cox regression analysis. **(B)** The distribution plot of the LASSO coefficient. Twelve variables were retained when log-transformed lambda equal to -3.31. **(C)** Twelve variables were retained when the partial likelihood deviation reached the minimum (Log Lambda = -3.31). **(D)** The Forest plot showed the coefficient, *p*-value, HR, and 95%CI of 3 prognosis-related TIR-lncRNAs calculated by multivariate Cox regression analysis.

To further explore the relationship between TIR-lncRNAs and the prognosis of RCC patients, we constructed a prognostic model for RCC. Univariate Cox regression was performed to screen prognosis-related TIR-lncRNAs and 62 prognosis-related TIR-lncRNAs with *p* < 0.05. The forest plot showed the *p*-value, hazard ratio (HR), and 95% confidence interval (CI) of prognosis-related TIR-lncRNAs (Figure 7A, two lncRNAs were not shown in Figure 7A because they had large 95%CI values, Supplementary Table S7). Subsequently, LASSO regression analysis was performed to prevent the overfitting of the prognostic signature. Twelve prognosis-related TIR-lncRNAs were identified when the log-transformed lambda equal to -3.31 (Figures 7B,C). Finally, using stepwise multiple Cox regression analysis, three TIR-lncRNAs were identified and used for modeling. The coefficient, *p*-value, HR, and 95% CI values of the TIR-lncRNAs involved in the risk model are shown in Figure 7D. The risk score for each patient was calculated based on the coefficient and log2-transformed TPM of TIR-lncRNAs.

**FIGURE 8:**
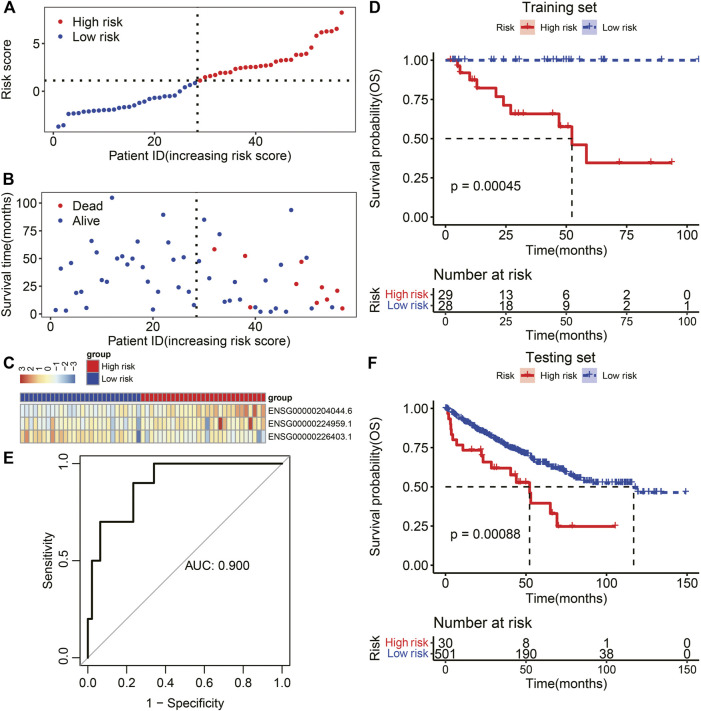
Evaluation and validation of TIR-lncRNA signature in RCC. **(A)** The risk curve of each sample was reordered by risk score. The red and blue dots represent high-risk and low-risk, respectively **(B)** Patients in the high-risk group showed higher mortality than those in the low-risk group. The red and blue dots represent death and survival, respectively. **(C)** Heatmap showed scaled expression levels of prognosis-related TIR-lncRNAs in the low-risk and high-risk groups. **(D)** Patients in the high-risk group (red) exhibited worse OS than those in the low-risk group (blue) in the training set. **(E)** The AUC values for forecasting OS status using the risk score in the training set. **(F)** Patients in the high-risk group (red) exhibited worse OS than those in the low-risk group (blue) in the testing set.

In the training set, RCC patients were divided into high-risk and low-risk groups according to the median risk score (Figure 8A). Patients in the high-risk group showed higher mortality rates than those in the low-risk group (*p* = 0.003, Figure 8B). The heatmap of the expression levels of the three TIR-lncRNAs revealed different expression levels between the high-risk and low-risk groups (Figure 8C). ENSG00000204044.6 and ENSG00000224959.1 were highly expressed in the high-risk group (Figure 8C), whereas ENSG00000226403.1 was highly expressed in the low-risk group (Figure 8C). K-M analysis revealed that RCC patients in the high-risk group exhibited worse overall survival (OS) than those in the low-risk group (*p* < 0.001, Figure 8D). The AUC of the risk score was 0.9 of OS (Figure 8E).

An independent dataset involving 531 samples was used to validate the TIR-related lncRNA signature. K-M analysis revealed that RCC patients in the high-risk group also exhibited worse OS than those in the low-risk group (*p* < 0.001, Figure 8F). These findings suggested that the TIR-lncRNA signature is efficient for predicting the prognosis of RCC.

### TIR-lncRNA Signature Was an Independent Prognostic Factor

**FIGURE 9:**
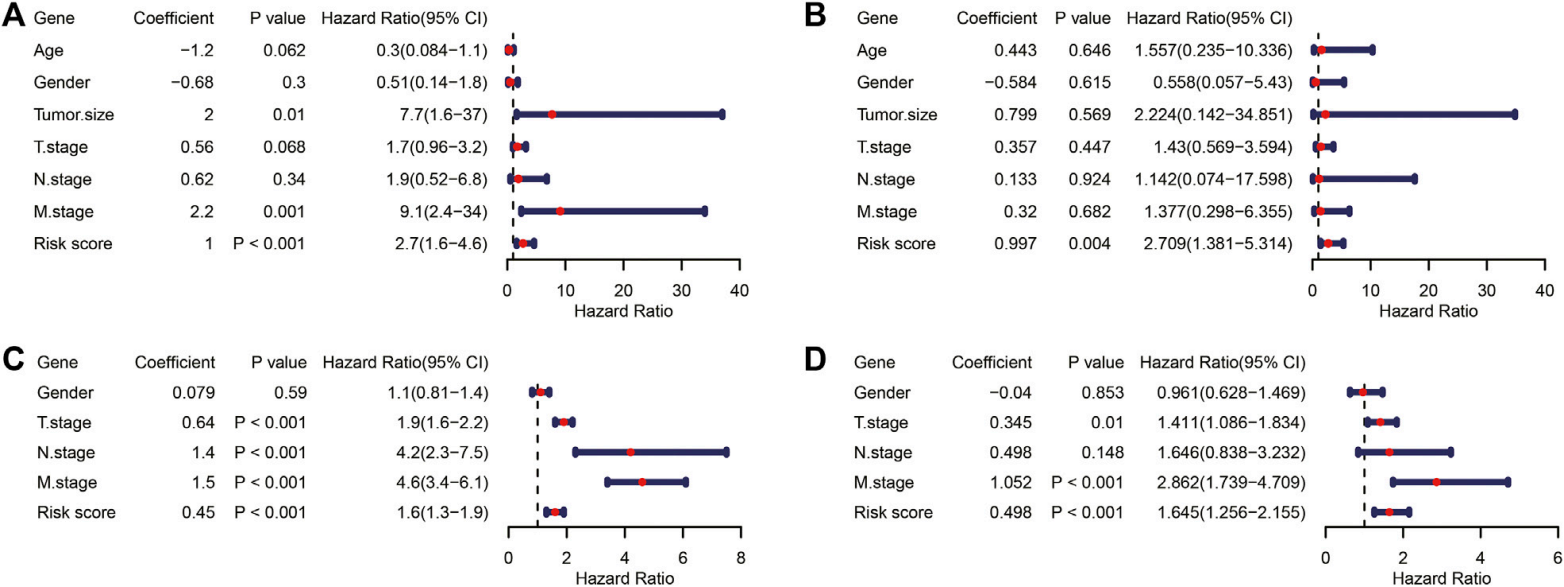
TIR-lncRNA signature was an independent prognostic factor for RCC. **(A,B)** The Forest plot showed the results of univariate Cox and multivariate Cox regression analyses in the training set. **(C,D)** The Forest plot showed the results of univariate Cox and multivariate Cox regression analyses in the testing set.

To explore whether the TIR-lncRNA signature was an independent prognostic factor for RCC, univariate and multivariate Cox regression analyses were performed to assess the independence of TIR-lncRNAs from other clinical factors, including age, gender, tumor size, and pathological stage in the training and testing sets, respectively. In the training set, the HR of the risk score and 95%CI were 2.7 and 1.6–4.6 in univariate Cox regression analysis (*p* < 0.001, Figure 9A), and 2.709 and 1.381–5.314 in multivariate Cox regression analysis (*p* = 0.004, Figure 9B), respectively. In the testing set, the HR of the risk score and 95%CI were 1.6 and 1.3–1.9 in univariate Cox regression analysis (*p* < 0.001, Figure 9C), and 1.645 and 1.256–2.155 in multivariate Cox regression analysis (*p* < 0.001, Figure 9D), respectively. These results suggested that the TIR-lncRNA signature was an independent prognostic factor for RCC.

### Construction of a Nomogram for Survival Prediction of RCC

To improve the model’s clinical practicability, a nomogram score system was constructed in the training set using the TIR-lncRNA signature, age, gender, tumor size, and pathological stage to predict 1-, 3-, and 5-years overall survival of RCC (Supplementary Figure S5). The nomogram’s concordance index (C-index) was 0.951, which increased the predictive power of OS compared with the TIR-lncRNA signature (C-index = 0.929).

## Discussion

In this study, immune-related lncRNA landscape was constructed, and 241 TIR-lncRNAs were functionally characterized, three of which were identified as a novel TIR-lncRNA signature for predicting the prognosis of RCC. First, raw transcriptomic data from the GEO database were used to identify novel lncRNAs. Subsequently, by comparing tumors with controls, we calculated TR-lncRNAs, TR-mRNAs, and TR-miRNAs. Then, an unsupervised clustering algorithm was utilized to classify RCC patients into different immune groups based on the infiltration level of immune cells. TIR-lncRNAs and TIR-mRNAs were identified by comparing the immune-high group with the immune-low group. A lncRNA/miRNA/mRNA ceRNA network based on miRNA-lncRNA and miRNA-mRNA pairs was constructed. In addition, a large part of TIR-lncRNAs were detected in urinary samples from RCC patients. Finally, three prognosis-associated TIR-lncRNAs were identified. To evaluate and validate the predictive ability of the prognostic signature, RCC patients were classified into high-risk and low-risk groups; patients in the high-risk group had worse OS than those in the low-risk group, with an AUC value of 0.9.

Patients were classified into three clusters based on the infiltration score of immune cells in each patient. However, to obtain immune-related lncRNAs, we only compared the immune-high group with the immune-low group. The immune-middle group was not used to calculate immune-related lncRNAs. Compared with the immune-low group, the immune-high group showed a significantly higher degree of infiltration of immune-cell types (Figure 4D, Supplementary Figure S2). However, the immune-middle group showed fluctuations in some immune cells (Supplementary Figure S2). For example, compared with the immune-high group, the immune-middle group exhibited a significantly larger number of immature dendritic cells, natural killer cells, effector memory CD4 T cells, immature B cells, activated CD4 T cells, memory B cells, and T-helper cell type 17. These results suggested that the immune-middle group was not suitable to identify immune-related lncRNAs.

Recent studies have focused on N6-methyladenosine (m6A)-, glycolysis-redox-, or immune-related lncRNA signature for predicting the prognosis of RCC. Yu et al. identified an m6A-related lncRNA signature for predicting the prognosis of RCC, with an AUC value of 0.80 (Yu et al., 2021). Ma et al. identified a glycolysis-related lncRNA prognostic signature for RCC and the AUC value was 0.82 (Ma et al., 2021). Dong et al. identified a redox-related lncRNA signature of RCC and the AUC value was 0.82 (Qi-Dong et al., 2020). Sun et al. constructed an immune-related lncRNA pair signature of RCC and the AUC value was 0.76 (Sun et al., 2021). In our prognostic model, we constructed a tumorigenesis-related and immune infiltration-related lncRNA signature for predicting the prognosis of RCC, with an AUC value of 0.9. This value is higher than those of previous prognosis models, supporting that our model is more efficient in predicting the prognosis of RCC.

Our study had some limitations. On the one hand, molecular-levels analyses are needed to further validate novel lncRNAs. On the other hand, the mechanism of TIR-lncRNAs in regulating protein-coding genes involved in RCC immunity are need to be further explored.

## Data Availability

The datasets presented in this study can be found in online repositories. The names of the repositories and accession number(s) can be found in the article.

## Supplementary Material

Supplementary Figure S1 Identification of TR-mRNAs by comparing tumors with controls in RCC. **(A)** Histogram of TR-mRNAs number in three GEO datasets. **(B)** Upset plots of the distribution of upregulated mRNAs in each dataset. **(C)** Upset plots of the distribution of downregulated mRNAs in each dataset.

Supplementary Figure S2 Comparisons among the immune-high group, the immune-middle group, and the immune-low group for the other 21 immune-cell types.

Supplementary Figure S3 Circos plot showed the distribution of TIR-lncRNAs on each chromosome. Upregulated TIR-lncRNAs were shown in red, while downregulated TIR-lncRNAs were shown in blue.

Supplementary Figure S4 The volcano plot showed TR-miRNAs by comparing tumors with controls in RCC. The red color represented upregulated miRNAs and the blue color represented downregulated miRNAs.

Supplementary Figure S5 The nomogram developed in the training set for predicting 1-, 3-, and 5-year survival of RCC patients.
