# Supplementary figures and images for: Systematic Investigation of Immune-Related lncRNA Landscape Reveals a Potential Long Non-Coding RNA Signature for Predicting Prognosis in Renal Cell Carcinoma
Source: Front Genet. 2022 Jul 4;13:890641. doi: 10.3389/fgene.2022.890641 (PMC9289211; doi:10.3389/fgene.2022.890641)

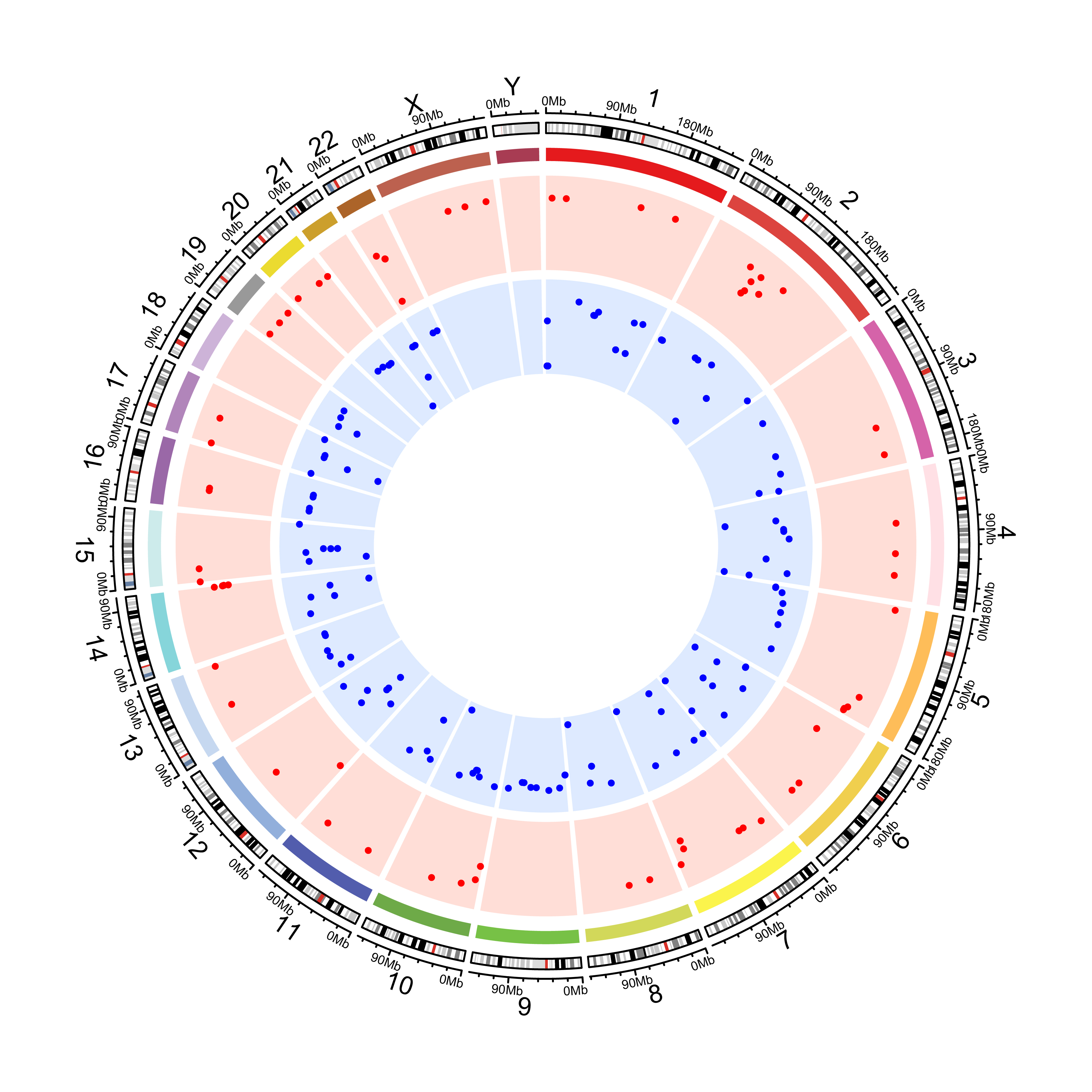

Supplement: Supplementary file 1 [file Image3.jpg]

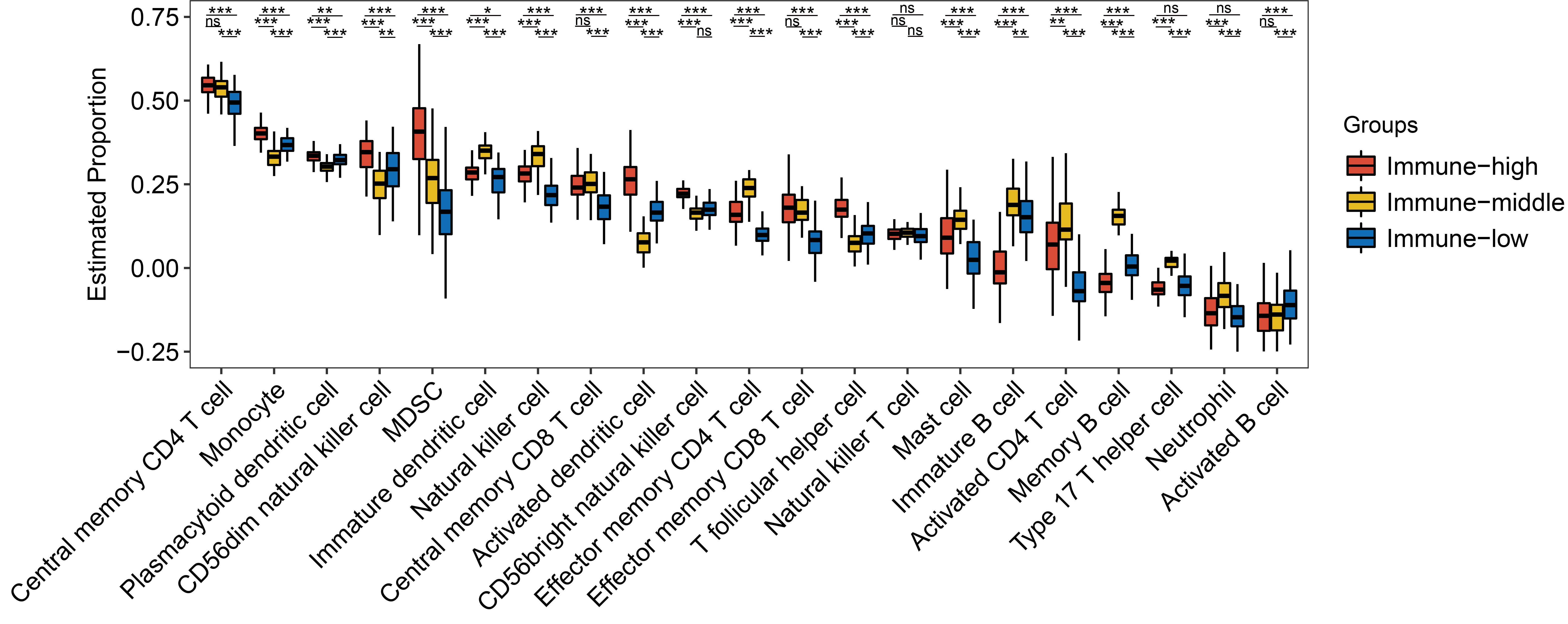

Supplement: Supplementary file 2 [file Image2.jpg]

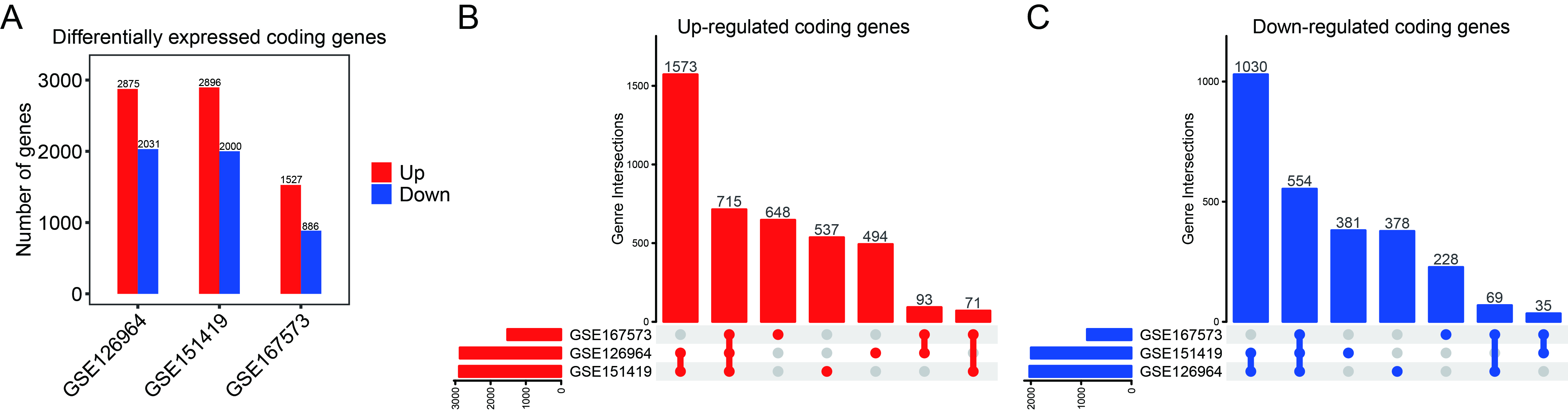

Supplement: Supplementary file 3 [file Image1.jpeg]

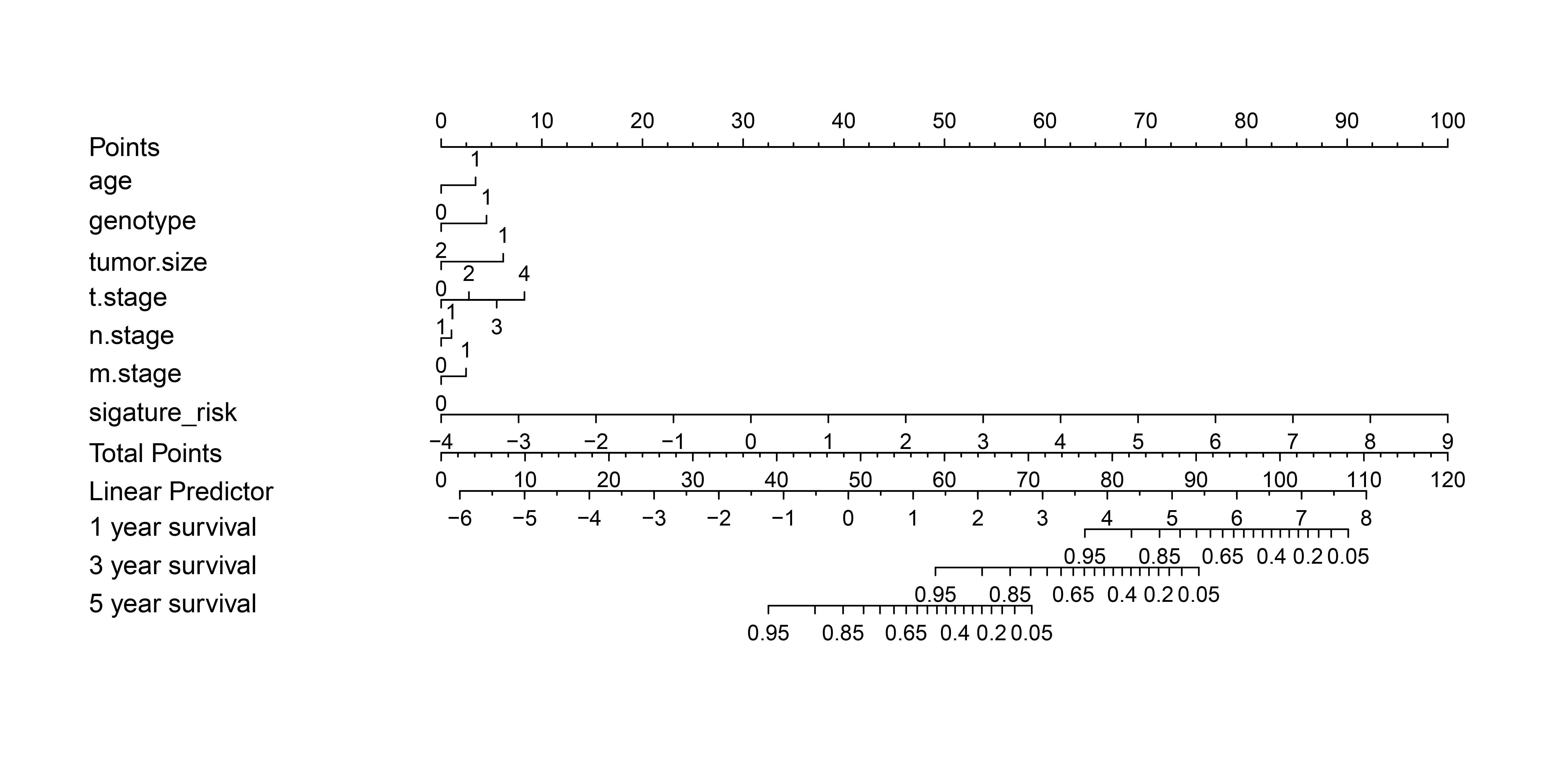

Supplement: Supplementary file 4 [file Image5.jpeg]

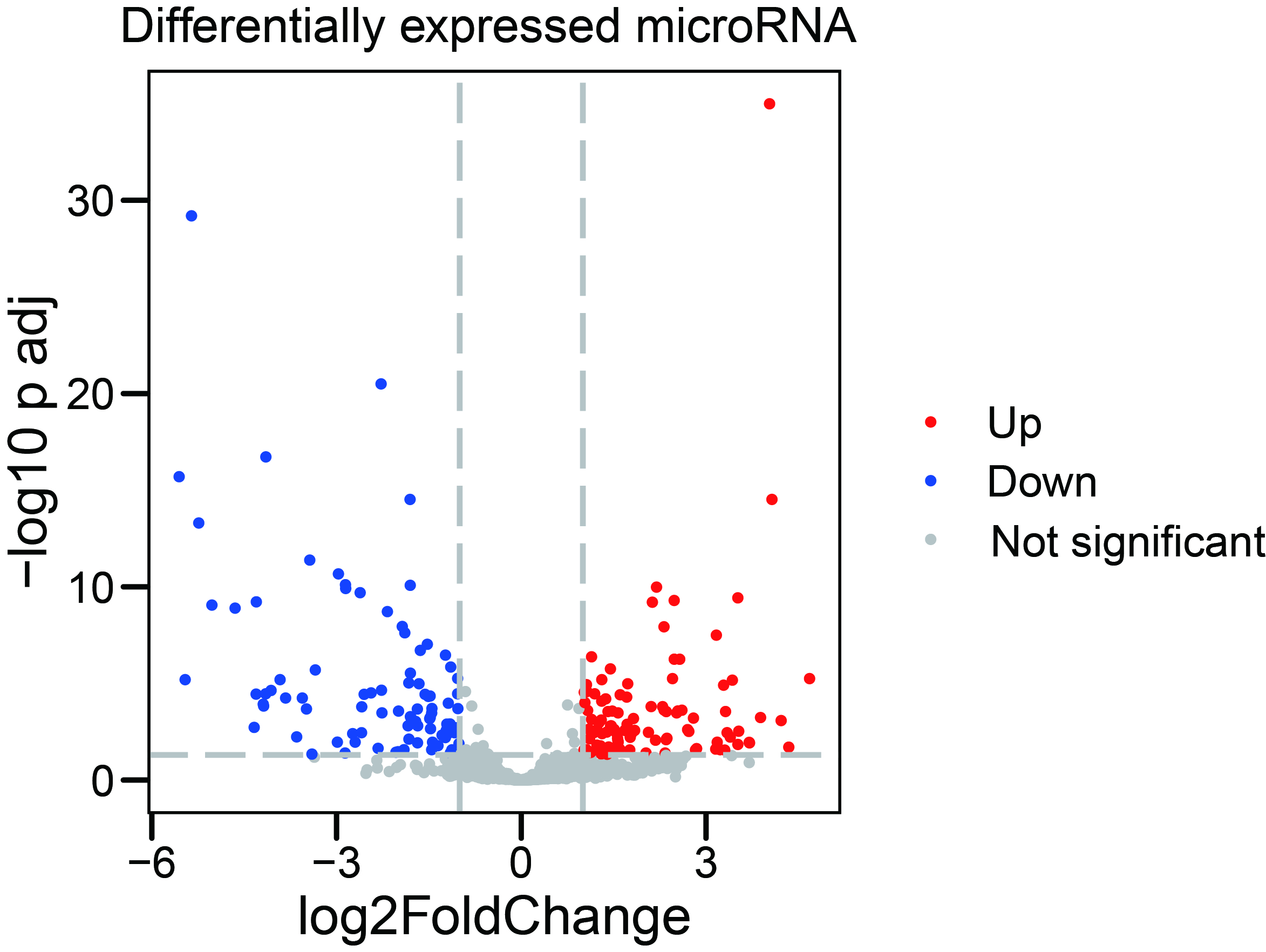

Supplement: Supplementary file 5 [file Image4.jpg]
